# Size- and Interface-Constrained Tensile Behavior of Ti/Ni Polycrystalline Nanolaminates: Insight from Molecular Dynamics

**DOI:** 10.3390/nano16100588

**Published:** 2026-05-12

**Authors:** Mengjia Su, Lanting Liu, Wei Hu, Qiong Deng

**Affiliations:** 1 School of Aeronautics, Northwestern Polytechnical University, Xi’an 710072, China; 2 National Key Laboratory of Strength and Structural Integrity, Xi’an 710072, China; 3 School of Science, Chang’an University, Xi’an 710064, China; liult@chd.edu.cn; 4 School of Flight Technology, Jiangxi Flight University, Nanchang 330088, China; 000570@jxfu.edu.cn; 5 Key Laboratory of Low Altitude Geographic Information and Air Route of Jiangxi Education Institutes, Nanchang 330088, China

**Keywords:** Ti/Ni polycrystalline nanolaminate, grain size, layer thickness, interface, constrained plastic deformation

## Abstract

Metallic nanolaminates (MNLs) exhibit excellent mechanical properties due to unique modulation and interface structures. However, the correlation between the deformation of nanostructures and the mechanical behavior of the materials remains inadequately elucidated. Molecular dynamics method is performed to investigate coupled effect of grain size (*d* = 7.5~25.0 nm) and layer thickness (*λ* = 1.31~15.15 nm) on the tensile behavior of Ti/Ni polycrystalline nanolaminates (PNLs). A plastic co-deformation mechanism involving crystalline phases, interface, and grain boundary under strong size and interface constraints is discovered. The dominant plastic deformation in Ti layer is size-independent HCP-BCC-HCP phase transformation. Dislocations propagation in Ni layer shifts with increasing layer thickness, which manifests as extended dislocations sliding, interaction between moving dislocations and interface dislocations, respectively. When grain sizes or layer thicknesses are small, interface migration, grain boundary diffusion, and grain boundary migration become prominent plastic deformation carriers. The coordinating effect of grain boundary and interface on deformations of different nanostructures endows materials with relatively favorable plastic properties. Moreover, a dimensionless parameter *d*/*λ* accounting for grain morphology and interface structure is found to predict the variations in flow stresses and characterize the dominating plastic deformation mechanisms of the stretched Ti/Ni PNLs.

## 1. Introduction

Metallic nanolaminates (MNLs) are layered composites formed by depositing two or more different metals and alloys alternately. And the individual layer thickness of the MNLs is on the scale of 1–100 nm [1,2]. Owing to unique layered structures and interface structures, MNLs exhibit excellent optical properties [3], electromagnetic properties [4], friction resistance [5], thermal stability [6], and radiation resistance [7,8]. In addition to excellent physical and chemical properties, MNLs also possess superior mechanical properties [1,9,10,11]. Thus, MNLs become promising candidates for applications in flexible electronics and micro/nano-electromechanical systems (MEMS/NEMS). The design flexibility also makes them ideal materials for investigating the mechanical behaviors of the nanomaterials.

Extensive experimental and simulation results indicate that the mechanical properties of face-centered cubic/face-centered cubic (FCC/FCC) and body-centered cubic/face-centered cubic (FCC/BCC) nanolaminates are closely related to their modulation structures and interface properties [12,13,14,15,16]. Moreover, the strength/hardness of isopachous nanolaminates show different trends with variations in layer thickness [12,15]. Interface plays a vital role in the deformations of nanolaminates by hindering or facilitating dislocations propagation. Moreover, interface also provides regions for interaction between interface dislocations and various plastic deformation carriers within the crystal [12]. Apart from the heterogeneous phases and interfaces, MNLs also contain a large number of grain boundaries (GBs), interface transition zones, and premixed layers. These nanostructures indeed affect the mechanical behavior of MNLs [17,18,19]. However, most established theories focus solely on the interaction between the intra-layer plastic deformation carriers and interfaces, without considering the influence of GB, phase boundary and other nanostructures on the mechanical behavior of the materials.

The MNLs prepared by vapor deposition methods exhibit the columnar polycrystalline morphology along the growth direction. And the layer thickness is typically equal to the grain size at this condition [20]. Therefore, both layer thickness and grain size jointly affect mechanical properties and plastic deformation mechanisms of MNLs. Compared to the extensive research on the effect of the layer thickness, there is a significant lack on how layer thickness and grain size jointly affect the mechanical behavior of the nanolaminates. Only Zhu et al. [21,22] and Fu et al. [23] investigated the coupled effects of grain size and layer thickness on the mechanical behavior of polycrystalline nanolaminates (PNLs) by molecular dynamics (MD) methods. Zhu et al. investigated the variations in the strength of Cu/Ag (FCC/FCC) PNLs with respect to layer thickness and grain size. They proposed a plastic deformation mechanism founded on dislocation theory and highlighted the role of grain boundary and interface during the tensile process [21,22]. Fu et al. investigated the variations in Cu/CoCrFeNi (FCC/FCC) PNLs with respect to characteristic dimensions and developed a model for quantitatively predicting flow stress by integrating various strengthening mechanisms [23]. However, there still remain significant doubts about whether the above conclusions can successfully illustrate the mechanical behavior of heterogeneous hexagonal close-packed/face-centered cubic (HCP/FCC) nanolaminates.

Due to differences in the slip systems and plastic deformation carriers, the phase interface can prevent plastic deformation carriers crossing from itself. The plastic deformations of HCP/FCC nanolaminates are more complex, which depends on the heterogeneous components and interface structure [24,25,26,27]. The authors have conducted an in-depth study of the mechanical behavior of Ti/Ni nanolaminates by MD methods. The results pointed out that the strength was profoundly governed by the modulation parameters, and the interfaces coordinated the plastic deformations by transforming from semi-coherent to incoherent regions during the tensile process [28,29]. As an extension of the aforementioned work, this study presents MD investigation of the coupled role of grain size (*d* = 7.5~25.0 nm) and layer thickness (*λ* = 1.31~15.15 nm) in governing the tensile behavior of Ti/Ni PNLs. Synergistic effects of feature size on flow stress and the plastic deformation mechanisms are elucidated. The yield stress is insensitive to the feature size, while plastic properties are enhanced at smaller *λ* and larger *d*. And the plastic deformations are governed by plastic co-deformation of different structures under strong size and interface constraints. The coordinating effect of grain boundary and interface on deformation endows the Ti/Ni PNLs with relatively favorable plastic properties. A dimensionless parameter (*d*/*λ*) accounting for grain morphology and interface structure are discovered to effectively characterize the controlling factors of different deformation mechanisms in HCP/FCC nanolaminates. These findings provide new fundamental insights and a design parameter for engineering high-performance metallic nanolaminates with tailored mechanical properties.

## 2. Materials and Methods

**Figure 1:**
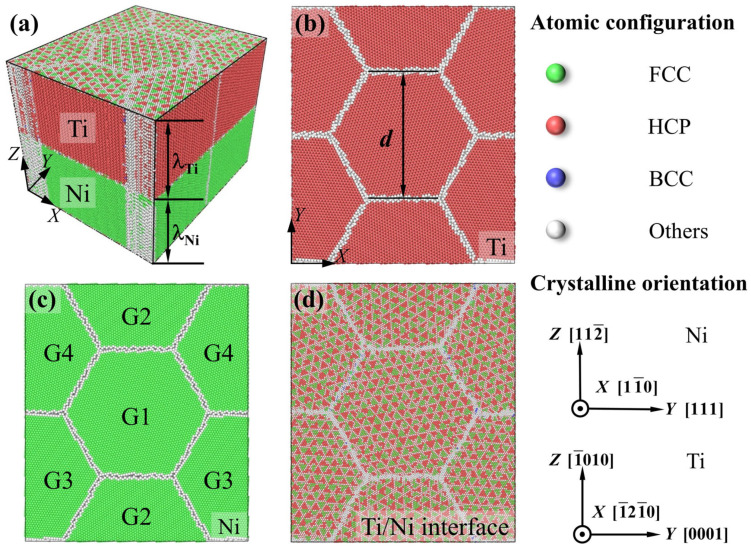
Schematic of Ti/Ni polycrystalline nanolaminates. (**a**) Isopachous Ti/Ni polycrystalline nanolaminates, (**b**) Atomic configuration of Ti layer, (**c**) Atomic configuration of Ni layer, (**d**) Atomic configuration of Ti/Ni interface. G1 to G4 are four grains with different crystalline orientations along Z-direction. Green, red, blue and light gray atoms represent FCC, HCP, BCC and other atoms identified by CNA method.

Figure 1 shows the atomic configurations of isopachous Ti/Ni PNL with each sublayer containing four columnar grains. The reason for selecting the established model is that the grains exhibit the columnar distribution along growth direction during the vapor deposition [30,31]. As shown in Figure 1a, the thicknesses of Ti and Ni layers were maintained equally, i.e., $\lambda_{\mathrm{Ni}}\;=\;\lambda_{\mathrm{Ti}}$, which ensured a ratio of 1:1. Six kinds of grain sizes ranging from 7.5 to 25.0 nm, and seven kinds of layer thicknesses ranging from 1.31 to 15.15 nm were chosen to investigate the coupling size effect on the tensile behavior of Ti/Ni PNLs. Thus, the total dimensions of the diverse samples range from $12.99\times 15.00\times 26.00{\;\mathrm{nm}}^{3}$ to $43.30\times 50.00\times 30.10{\;\mathrm{nm}}^{3}$. The initial crystalline orientations of distinct components were also listed on the right side of Figure 1.

Figure 1b and Figure 1c demonstrate that Ti and Ni sublayers exhibit a pronounced {0001} and {111} texture along the Z-direction, respectively. This preferential orientation arises because atoms tend to stack along close-packed planes during vapor deposition [30,31]. Additionally, the initial grains were rotated counterclockwise by 11.25° (G1), 33.75° (G2), 56.25° (G3) and 78.75° (G4) along the Z-axis to generate new simulated models and avoid the formation of special orientation relationships between diverse grains. Due to the different crystalline structures of the components, a semi-coherent Ti/Ni interface forms at the junction of matrix phases. As shown in Figure 1d, the FCC and HCP atoms at the Ti/Ni interface are arranged in a periodic pattern and separated by the misfit dislocations.

Interatomic potential is crucial for the accuracy of MD simulations. In present work, the Embedded Atom Method (EAM) potential proposed by Zhou and Wadley et al. [32,33] was employed to simulate atomic interactions among Ti-Ti, Ti-Ni and Ni-Ni atoms. This potential had described the mechanical behaviors of the Cu/Ta [34], Cu/Ag [35], Ti/Al [36] and Ti/Ni [37] nanolaminates effectively. To assess the applicability of the potential for Ti and Ni, the lattice constants and cohesive energies for both components, and generalized stacking fault energy (GSFE) curves for Ni had been validated in Supplementary Material. It can be seen from Figures S1 and S2 and Table S1 that the potential can accurately reproduce these essential properties. In addition, the Velocity-Verlet integration algorithm was utilized to solve the kinetic parameters of all particles [38]. The periodic boundary conditions (PBCs) were applied in all directions to eliminate the surface effects and focused on the role of internal nanostructures during the tensile process. The temperature was set as 300 K, and the Nose–Hoover method was used to maintain a constant temperature of the system [38]. The timestep was selected as 2 fs (10^−15^ s) in all simulations. Energy minimization was performed by utilizing the conjugate gradient method to optimize atomic positions. Since energy of the sample generated by the computer was significantly higher than that of the actual material, the system must be relaxed in isothermal–isobaric (NPT) ensemble for 120 ps (10^−12^ s) to obtain the stable configuration. Subsequently, the tension loading was applied by stretching the atoms with a constant strain rate of $\dot{\varepsilon }\;=\;5\times 10^{8}\;s^{-1}$ along the Y-direction. And the total strain was set as 0.08. To validate the effectiveness of the simulation method for studying the tensile behavior of Ti/Ni PNLs, three replicated tests for Ti/Ni PNL with *d* = 7.5 nm and *λ* = 1.31 nm had been performed in Supplementary Material. It can be seen from Figures S3 and S4 that the algorithm and simulation method in this work can effectively reproduce the equilibrium and tensile properties of Ti/Ni polycrystalline nanolaminate. Different from the macroscopic stress, the virial theorem was used to calculate the atomic stress, and the stress of the system was obtained by summing all atomic stresses [39]. All the simulation work was performed by using the large-scale atomic/molecular massively parallel simulator (LAMMPS) [40]. The common neighbor analysis (CNA) method [41] was utilized to analyze the different structures, as well as to examine defects within the crystalline structures. In Figure 1, green, red, blue, and light gray atoms represent FCC, HCP, BCC, and other atoms. Additionally, the dislocation extraction algorithm (DXA) [42] was applied to identify dislocation types in specific crystalline structures. All aforementioned structure analysis and defect identification methods were implemented by using the visualization software of the open visualization tool (OVITO pro 3.15.4) [43].

## 3. Results

### 3.1. Tensile Properties of Ti/Ni Polycrystalline Nanolaminates

**Figure 2:**
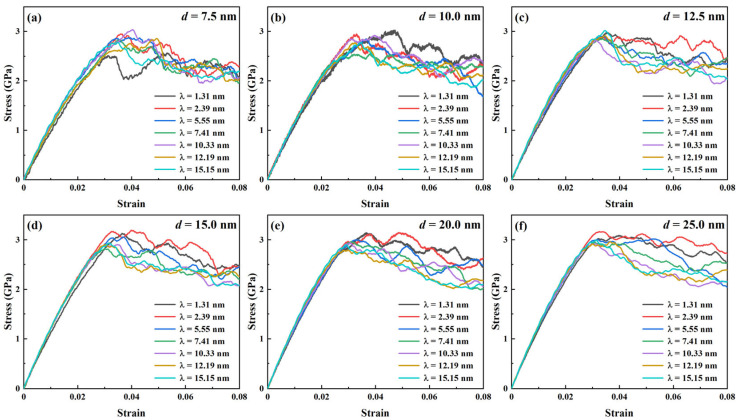
Stress–strain curves of Ti/Ni polycrystalline nanolaminates with different grain sizes and layer thicknesses. (**a**) *d* = 7.5 nm, (**b**) *d* = 12.5 nm, (**c**) *d* = 12.5 nm, (**d**) *d* = 15.0 nm, (**e**) *d* = 20.0 nm, (**f**) *d* = 25.0 nm.

Figure 2 shows the stress–strain curves for Ti/Ni PNLs with different grain sizes and layer thicknesses. As shown in Figure 2a–f, there are no noticeable stress drops for all Ti/Ni PNLs after the initial elastic stage. Instead, the curves fluctuate at relatively high stresses or decline slightly. This fact indicates that Ti/Ni PNLs possess good plastic properties during the tensile process. Moreover, it is obvious that variations in grain size and layer thickness rarely affect the yield stress of Ti/Ni PNL except the sample with *d* = 7.5 nm and *λ* = 1.31 nm. Here, the yield stress is defined as the point where the linear elastic region transitions into the plastic flow region, i.e., the minimum stress required to induce further plastic deformations [44]. Most Ti/Ni PNLs exhibit yield stresses around 3 GPa, whereas the sample with *d* = 7.5 nm and *λ* = 1.31 nm shows a lower value of approximately 2.5 GPa. The results in Figure 2 suggest that changes in size seldom affect yield stress of the Ti/Ni PNL.

**Figure 3:**
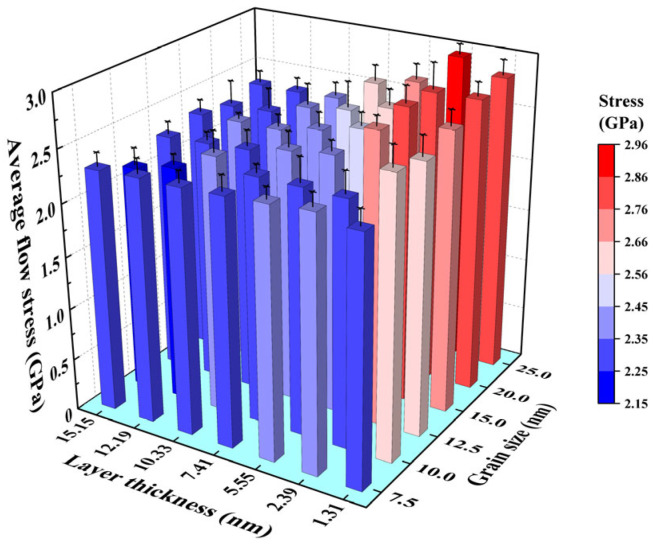
Average flow stresses of Ti/Ni polycrystalline nanolaminates with different grain size and layer thickness. Error bars indicate the standard deviation.

On the contrary, the flow stresses show different trends with changing grain size and layer thickness. Flow stress in this work corresponds to average stress ranging from 0.04 to 0.08 of the stress–strain curves. Figure 3 illustrates the trends of average flow stresses of Ti/Ni PNLs with the variations in layer thickness and grain size. When the grain size is constant, the average flow stresses increase with decreasing the layer thickness. When the layer thickness is constant, changes in grain size have little effect on the flow stresses with layer thicknesses ranging from 10.33 to 15.15 nm. As the layer thickness further decreases, i.e., *λ* ≤ 7.41 nm, the flow stresses decrease with increasing the grain size. Overall, when the grain size and layer thickness are located in the upper-right corner of Figure 3, the sample possesses the higher flow stress. In other words, the Ti/Ni PNLs possess sustained high-flow stress under the conditions of smaller layer thickness and larger grain size.

The average flow stress provides a better reflection of the atomic configuration evolutions during the tensile process. And the plastic deformation mechanisms of Ti/Ni PNLs are significantly dominated by the grain size and layer thickness. The atomic configuration evolutions of Ti/Ni PNLs under different characteristic parameters will be discussed in the following sections.

### 3.2. Atomic Configuration Evolutions of Ti/Ni Polycrystalline Nanolaminates

**Figure 4:**
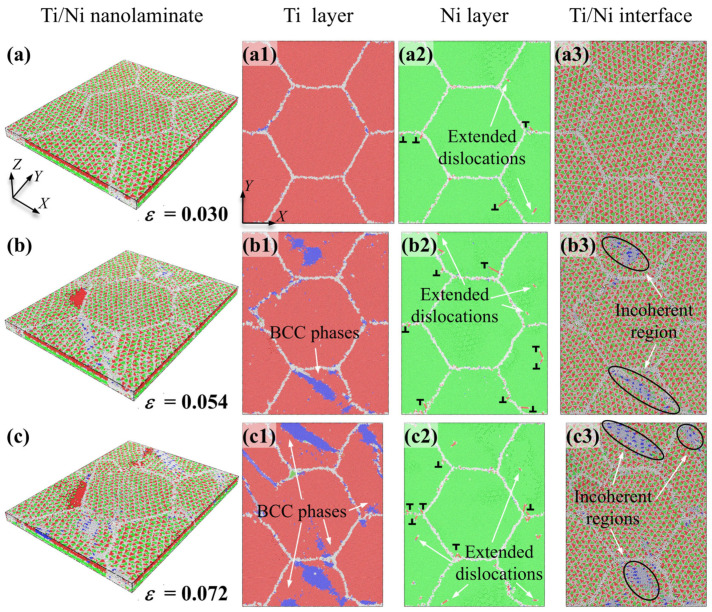
Atomic configuration evolutions of Ti/Ni polycrystalline nanolaminates with *d* = 25 nm and *λ* = 1.31 nm. (**a**) *ε* = 0.030, (**b**) *ε* = 0.054, (**c**) *ε* = 0.072. The subfigures show the atomic configurations of Ti sublayer (**a1**–**c1**), Ni sublayer (**a2**–**c2**) and Ti/Ni interface (**a3**–**c3**) in the X-Y plane, respectively.

Figure 4a–c shows the atomic configuration evolutions of Ti/Ni PNL with *d* = 25.0 nm and *λ* = 1.31 nm. Additionally, the subfigures illustrate the atomic configurations of the Ni sublayer, Ti sublayer, and Ti/Ni interface in the X-Y plane under identical strain, enabling direct observation of plastic deformation within each individual layer.

After the elastic stage, the plastic deformations first occur at the Ni layer. As shown in Figure 4a, patrial dislocations nucleate in the regions near grain boundaries at *ε* = 0.030 and propagate along specific directions within different Ni grains. Leading dislocations and trailing dislocations nucleate rapidly and slide along the identical plane, which leads to formation of extended dislocations, as indicated by the arrow in Figure 4(a2). Owing to the limited space, Ti/Ni interface confines the moving dislocations to propagate within the Ni layer and slip perpendicular to the interfaces. This observation aligns with simulations of uniaxial tensile deformation in Cu/Ag PNLs, which indicates that extended dislocation slip operates as a distinct plastic deformation mechanism under the constraint of extremely thin layers [21]. However, there are no significant plastic deformations observed in the Ti sublayer and Ti/Ni interface at the consistent strain. When the tensile strain reaches 0.053, BCC-Ti atoms turn to appear near the grain boundaries, as pointed out by the arrows in Figure 4(b1). The phase transformation is accompanied by structure evolution at the Ti/Ni interface. Specifically, interfaces promoting formation of BCC atoms progressively evolve into incoherent regions, highlighted by the ellipse in Figure 4(b3). While dislocations emit continuously from grain boundaries and slip in the form of extended dislocations in Ni layer. Upon further loading, the rise in the number of BCC-Ti atoms accelerates the transformation of semi-coherent regions into incoherent regions. Plastic deformations in Ni layer are also dominated by the propagation and interaction of extended dislocations, as displayed in Figure 4c. Apart from the grain interiors and interface, Figure 4 also denotes that grain boundaries in Ti layer also participate in plastic deformations via GB migrations. Therefore, the plastic deformations of Ti/Ni PNL with *d* = 25.0 nm and *λ* = 1.31 nm involve the propagation of extended dislocations in Ni layer, HCP to BCC phase transformations and GB migrations in Ti layer, and the transformation from semi-coherent regions into incoherent regions of the Ti/Ni interface.

**Figure 5:**
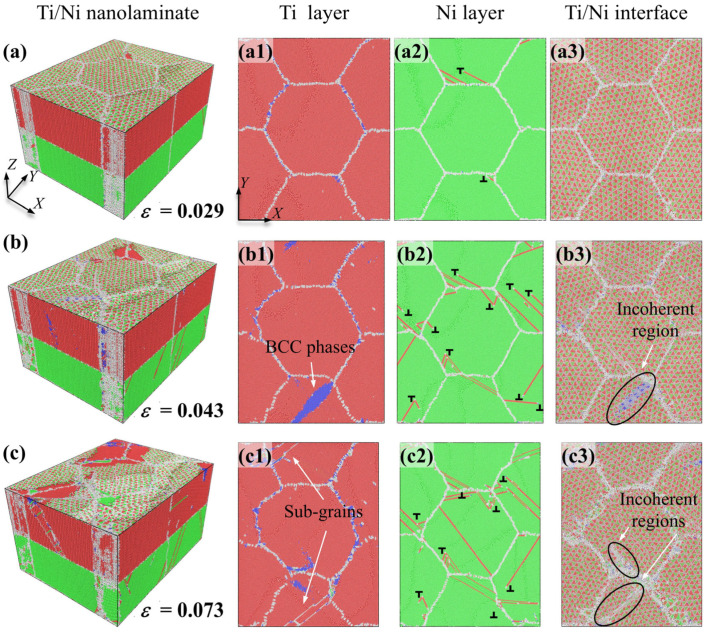
Atomic configuration evolutions of Ti/Ni polycrystalline nanolaminates with *d* = 25 nm and *λ* = 15.15 nm. (**a**) *ε* = 0.029, (**b**) *ε* = 0.043, (**c**) *ε* = 0.073. The subfigures show the atomic configurations of Ti sublayer (**a1**–**c1**), Ni sublayer (**a2**–**c2**) and Ti/Ni interface (**a3**–**c3**) in the X-Y plane, respectively.

When the layer thickness increases to 15.15 nm, the plastic deformations in Ni layer differ from former one. As shown in Figure 5a, patrial dislocations emit from grain boundaries at *ε* = 0.029. Due to the sufficient space for dislocations sliding, these dislocations propagate along specific planes in Ni layer, which results in formation of the HCP stacking faults within the Ni grains. The appearance of stacking faults leads to the disruption of periodically arranged Ti/Ni interfaces. Plastic deformations in Ti layer do not initiate at *ε* = 0.029, only a small number of BCC atoms appear near the grain boundaries. When the strain reaches 0.043, BCC-Ti rapidly nucleates at the grain boundaries and diffuses within the grains. And the plastic deformation in Ni layer is dominated by interactions between partial dislocations. In addition, some dislocations are blocked or absorbed by the interface, resulting in the formation of local incoherent regions at the interfaces, as displayed in Figure 5b. Under the greater strain, BCC-Ti atoms progressively revert to the HCP structure, accompanied by the nucleation of new sub-grains with distinct crystalline orientations. And GB migration in Ti layer can also be observed at *ε* = 0.073. In the meantime, the incoherent regions at the Ti/Ni interface also expand with the help of external strains. Therefore, the plastic deformations of Ti/Ni PNL with *d* = 25.0 nm and *λ* = 15.15 nm involve the propagation and interaction of partial dislocations in Ni layer, HCP-BCC-HCP phase transformations and GB migration in Ti layer, and the transformation from semi-coherent regions to incoherent regions of Ti/Ni interface.

**Figure 6:**
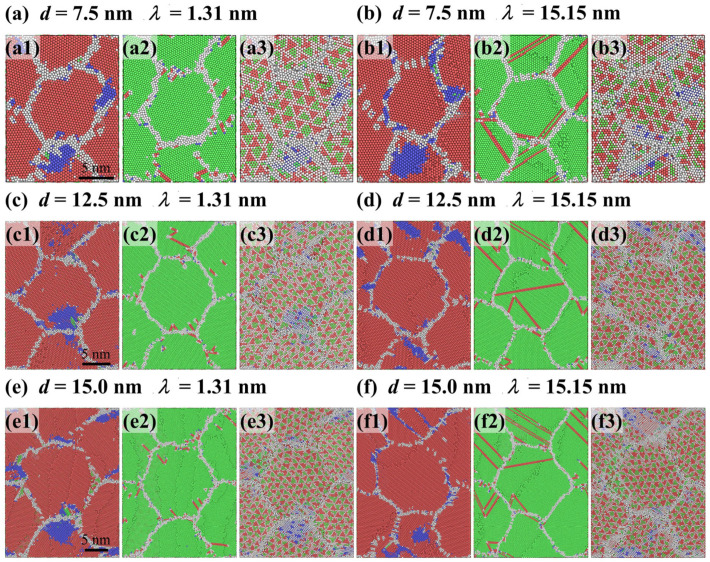
Atomic configuration evolutions of Ti/Ni polycrystalline nanolaminates with different grain sizes and layer thicknesses at *ε* = 0.072. (**a**) *d* = 7.5 nm and *λ* = 1.31 nm, (**b**) *d* = 7.5 nm and *λ* = 15.15 nm, (**c**) *d* = 12.5 nm and *λ* = 1.31 nm, (**d**) *d* = 12.5 nm and *λ* = 15.15 nm, (**e**) *d* = 15.0 nm and *λ* = 1.31 nm, (**f**) *d* = 15.0 nm and *λ* = 15.15 nm. The subfigures show the atomic configurations of Ti sublayer (**a1**–**f1**), Ni sublayer (**a2**–**f2**) and Ti/Ni interface (**a3**–**f3**) in the X-Y plane, respectively.

Further investigating the coupled effect of layer thickness and grain size on plastic deformation of Ti/Ni PNLs, Figure 6 presents atomic configurations for different feature sizes at *ε* = 0.072. Compared with different configurations, partial dislocations tend to slip in the form of extended dislocations with *λ* = 1.31 nm, while dislocations slip along adjacent planes and interact with moving dislocations with *λ* > 1.31 nm. At the smaller grain sizes, grain boundaries in Ni layer can also contribute to plastic deformation through GB migration or diffusion. However, the extent of GB migration is inversely proportional to the grain size. While for the Ti layer, the grains always achieve plastic deformations via allotropic phase transformations. And the grain boundaries of Ti layer can also participate in plastic deformations by GB migration. In addition, the proportion of local incoherent regions at the Ti/Ni interface increases as grain size decreases under the same strain. It can be inferred from Figure 2, Figure 3, Figure 4, Figure 5 and Figure 6 that the initial plastic deformation in Ti/Ni PNL occurs at the grain boundaries, manifesting as the nucleation and propagation of dislocations. This observation also explains why the yield stress seldom varies with layer thickness. Under the constraint of interface, dislocation movements are constrained within the Ni layer and interact with the interface or grain boundaries by disparate ways. The interaction between dislocations and interface leads to changes in the interface structure, which leads to promoting the phase transformations and GB motion in Ti layer. Since plastic deformations occur in both components, Ti/Ni interface coordinates the overall plastic deformations by transforming from semi-coherent regions to incoherent regions.

## 4. Discussion

### 4.1. Phase Transformation in Ti Grain

**Figure 7:**
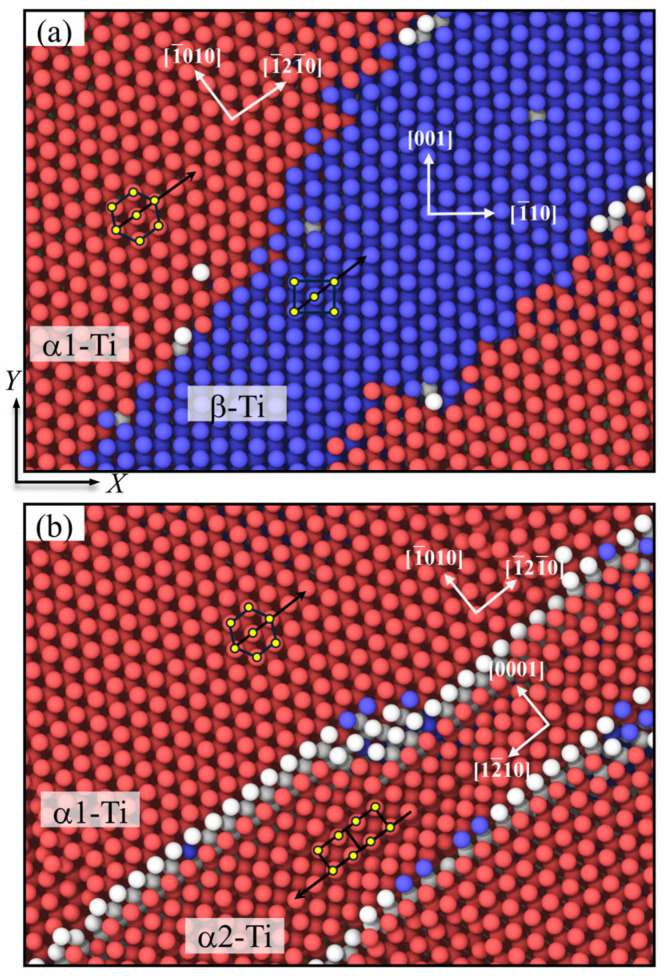
Grain orientation relationship of HCP-BCC-HCP phase transformation in Ti layer during the tensile process. (**a**) *ε* = 0.043, (**b**) *ε* = 0.073. The coordinates within different grains represent local coordinates in the specific crystals.

The atomic configuration evolutions of Ti/Ni PNLs indicate that the plastic deformations in Ti grain are the HCP-BCC-HCP phase transformation. This phase transition process is consistent regardless of feature size. Searching for more details, Figure 7 reveals the grain orientation relationships of phase transformation in Ti layer during the tensile process. For simplicity, the initial HCP phase is defined as α1-Ti, and BCC phase is defined as β-Ti, while the secondary HCP phase is defined as α2-Ti. It can be observed in Figure 7a that orientation relationships between α1-Ti and the newly formed β-Ti are ${\left\{0001\right\}}_{\alpha 1}||{\left\{110\right\}}_{\beta }$ and $<11\bar{2}0{>}_{\alpha 1}||<11\bar{1}{>}_{\beta }$, which is also called the Burgers orientation relationship [45]. The phenomena observed are consistent with observations when compressive loading is subjected along the prismatic plane of monocrystal Ti [46,47]. In present work, tension loading subjected parallel to the basal plane is equivalent to compression loading subjected perpendicular to the prismatic plane. This states that HCP-BCC phase transformation is commonly observed in deformed HCP-Ti metals. The transformation proceeds via shear on a {0001} plane concurrent with c-axis compression, ultimately establishing the <111> orientation in the new BCC lattice. Subsequently, the atoms achieve further deformation through atomic shuffle to fill the lattice vacancies and form the BCC phase ultimately.

Further increasing the tensile strain, the BCC atoms transform back into the HCP atoms. However, the crystalline orientations of the newly formed HCP crystal are different from the initial HCP phase. The orientation relationships between α1-Ti and α2-Ti are ${\left\{0001\right\}}_{\alpha 1}||{\left\{10\bar{1}0\right\}}_{\alpha 2}$ and $<11\bar{2}0{>}_{\alpha 1}||<11\bar{2}0{>}_{\alpha 2}$. The reason for the different orientations lies in the symmetry of HCP crystals. A single HCP grain can form six different BCC orientation variants, i.e., V1 toV6. Therefore, when a BCC grain transforms back to the HCP structure, the BCC grain randomly selects one or more of these variants with the help of external loads or grain boundary constraints. Each variant has the unique {110} plane and <111> direction. Selecting a disparate variant results in a rotation of basal plane and a-axis direction of HCP lattice, accomplishing crystalline reorientation in deformed HCP metals [45]. Based on the above findings, the phase transformation of HCP-BCC-HCP structures in Ti sublayer shows the crystalline orientation relationships of ${\left\{0001\right\}}_{\alpha 1}||{\left\{110\right\}}_{\beta }||{\left\{10\bar{1}0\right\}}_{\alpha 2}$ and $<11\bar{2}0{>}_{\alpha 1}||<11\bar{1}{>}_{\beta }||<11\bar{2}0{>}_{\alpha 2}$. Moreover, the macroscopic reorientation is achieved by selecting different variants, mediated at the atomic level by shear and shuffle according to the Burgers orientation relationship.

### 4.2. Plastic Deformations on Grain Boundary

**Figure 8:**
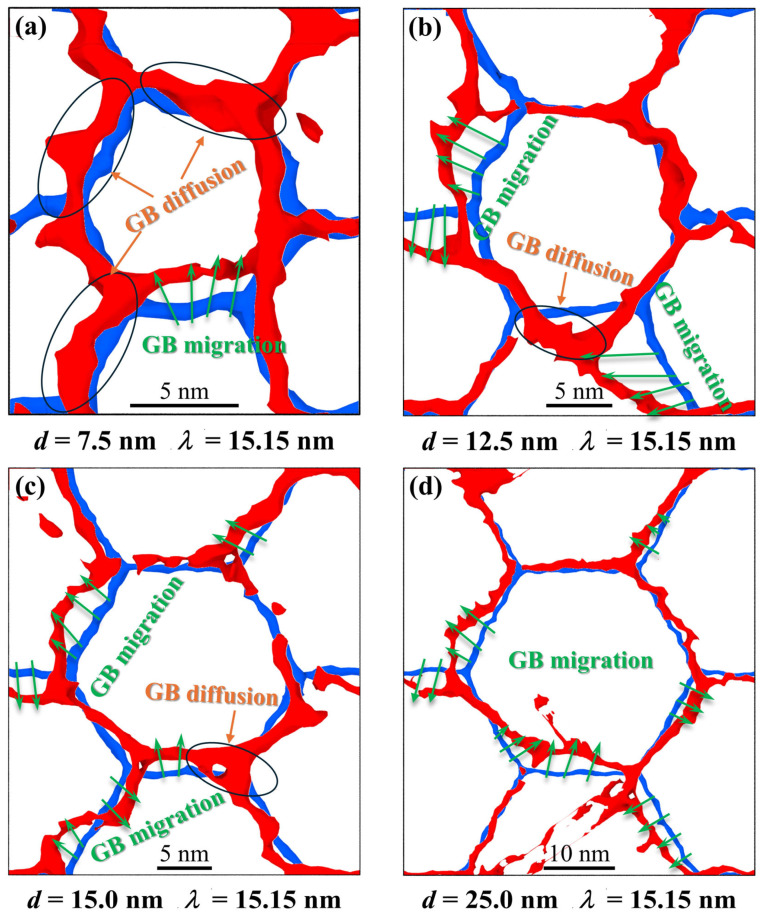
Grain boundary evolutions in Ti layer for Ti/Ni polycrystalline nanolaminates with different grain sizes and layer thicknesses. (**a**) *d* = 7.5 nm and *λ* = 15.15 nm, (**b**) *d* = 12.5 nm and *λ* = 15.15 nm, (**c**) *d* = 15.0 nm and *λ* = 15.15 nm, (**d**) *d* = 25.0 nm and *λ* = 15.15 nm. All atoms have been removed from the simulation box to better observe the deformation of the GBs. The blue configuration in the figure represents the GB morphology at *ε* = 0, while the red one is the configuration at *ε* = 0.072.

Atomic configuration evolutions from Figure 4, Figure 5 and Figure 6 prove that GBs in Ni layer can promote the nucleation of partial dislocations and absorb dislocations moving toward them. GBs with small sizes can also contribute to overall plastic deformation through migration or diffusion. And GB motions can be found in Ti layer regardless of grain size. In fact, the GB motions can coordinate plastic deformation between separate grains. Further analyzing how feature size affects the GB motions, Figure 8 shows the GB evolutions in Ti layer with different grain sizes and layer thicknesses. All atoms have been moved away from the simulation box to better observe the deformation of the GB in Figure 8. The blue configurations represent the morphologies of GB at *ε* = 0, while the red ones are the configurations at *ε* = 0.072. For the case to enable a direct comparative analysis of GB motions, the dimensions of the models are required to be maintained equally before and after deformation. This paper maps the model size at *ε* = 0.072 to the model size at *ε* = 0. This guarantees that the internal structure is also mapped, which allows for a more intuitive analysis of GB motions at different strains [43].

As shown in Figure 8a, the GB motions for the sample with *d* = 7.5 nm and *λ* = 15.15 nm contain GB diffusion and migration. Under these conditions, plastic deformations are mainly controlled by GB diffusion. As the layer thickness increases, the contribution of GB diffusion to the total plastic deformations gradually decreases. When the layer thickness reaches 25.0 nm, plastic deformations are fully dominated by GB migration. The observation reveals that these two types of GB motions are vital components for the overall plastic deformations of TI/Ni PNLs. Variations in grain size significantly affect the proportions and dominance of these two mechanisms during the tensile process. When multiple intragranular deformation carriers are hindered, GB migration is activated as a primary mechanism. This leads to concomitant changes in grain shape, size, and crystallographic orientation [48]. Rather than directly displacing grains, GB diffusion supports complementary deformation at grain boundaries, thus permitting stress accommodation without cracking [49]. The reduction in grain size significantly increases the proportion of GB diffusion and leads to the degradation of the migration, which finally alters the overall mechanical properties of Ti/Ni PNLs. This is one reason why the yield stress of PNL with *d* = 7.5 nm and *λ* = 1.31 nm is lower than that of other samples.

It can be observed from atomic configuration evolutions that there are relatively few plastic deformation carriers within the tiny grains. This is attributed to the small grain size inhibiting the further plastic deformation within the grains. At this stage, GBs account for a relatively large proportion of the material, and plastic deformations of GBs contribute significantly to the overall plastic deformations. As grain size increases, the proportion of plastic deformation carriers occurring within the grains gradually rises. Different plastic deformation carriers are continuously activated under the external loading, which results in the deformations of the grain interior. At this stage, GB migration helps to coordinate plastic deformations between different grains. The above results illustrate that GBs facilitate plastic deformations through diffusion or migration. The relative proportions of two deformation modes are dependent on the grain size. GB motions can coordinate plastic deformations among different grains. In particular, when grain sizes are small, GB motions contribute significantly to the overall plastic deformations of the material.

### 4.3. Plastic Deformations in Ti/Ni Interface

Metallic nanolaminates possess dense interfaces, which can exhibit superior mechanical properties than the conventional materials. Current theories propose that the exceptional strength of MNL is related to the obstruction of dislocations propagation at the interface. The interface acts as a barrier, constraining dislocation glide and promoting a transition from long-range slip to confined layer sliding. This mechanism significantly enhances strength of the material [50,51]. When the layer thickness reduces below several tens of nanometers, the plastic deformations no longer involve the dislocation-confined layer slip and dislocations crossing through interface. Interface itself starts to transform from passive barrier into a unit participating in plastic deformations. Thus, a series of plastic deformation carriers driven by interface are activated [52]. These plastic deformation carriers predominantly contain interface sliding [52,53,54], nucleation and propagation of interfacial dislocation [12,55], and interface migration [52].

**Figure 9:**
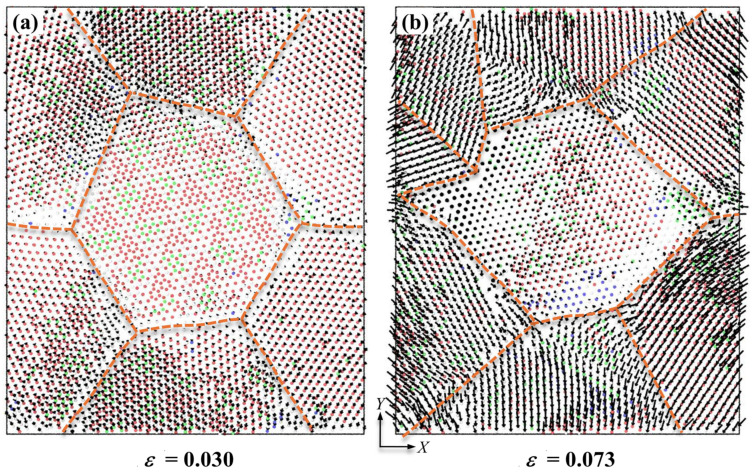
Atomic displacements of interfacial atoms in Ti/Ni polycrystalline nanolaminate with *d* = 7.5 nm and *λ* = 15.15 nm at different strains. (**a**) *ε* = 0.030, (**b**) *ε* = 0.073. The dashed lines in the figure are the intersection of grain boundaries and interface. The black arrows are used to represent the displacement vectors of interface atoms.

In present work, Ti/Ni interface achieves plastic deformations via interface migration, and the deformation becomes more pronounced as the feature size decreases. Figure 9 displays the displacement distribution of interfacial atoms at different strains, and the dashed lines represent the intersection of GBs and the interface. As shown in Figure 9a, the initial plastic deformation carriers originate at *ε* = 0.030. Interfacial atoms deviate slightly from their initial positions under shear stress, and displacement vectors are small at this strain. As the applied load gradually increases, a large number of interfacial atoms begin to deviate from their initial positions and migrate the larger displacements. When the tensile strain reaches 0.073, the interfacial atoms move significant displacements except the atoms in the central interface region. Furthermore, it can be observed that the distribution of displacements in different regions exhibits a distinct directionality, indicating that the interface migration can be achieved through atomic shuffle. This localized atomic movement can effectively relieve stress/strain concentration at the interface and coordinate plastic deformations of the adjacent components [56]. In addition, the GB migrations and formation of sub-grains in crystalline components are also discovered in Figure 9. Therefore, the interface migration and GB migration are coupled to promote the overall plastic deformations of the material. In summary, when the layer thickness of Ti/Ni PNLs ranges from tens of nanometers to a few nanometers, the interfaces begin to bear greater plastic deformations and coordinate plastic deformations between different phases through the coupled interface migration and GB migration.

### 4.4. Synergistic Effect of Grain Boundary and Interface on Dislocations Propagation in Crystalline Ni Layer

**Figure 10:**
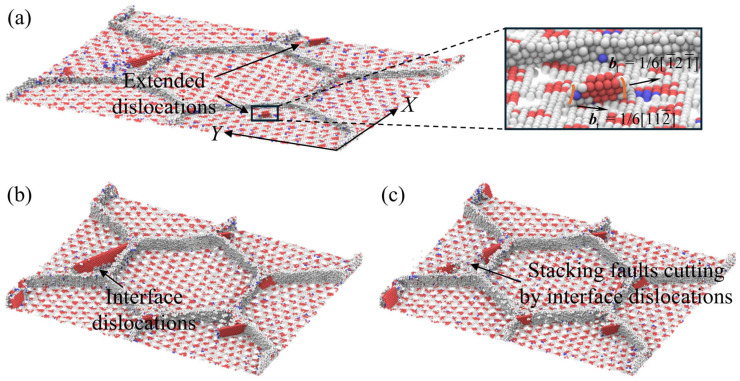
Dislocation propagations in Ni sublayer under grain boundary and interface constraints for the polycrystalline Ti/Ni nanolaminate with *d* = 25.0 nm. (**a**) *λ* = 1.31 nm, (**b**,**c**) *λ* = 2.39 nm. The upper interface and FCC atoms have been removed to better observe defect evolutions. The enlarged illustration shows Burgers vector of the extended dislocations. The yellow lines in enlarged illustration represent the leading dislocation and trailing dislocation, respectively.

Atomic configuration evolutions of Ti/Ni PNLs also indicate that dislocations propagation in Ni layers is controlled by both grain boundary and interface, which exhibits different deformation patterns with changing the feature size. Further investigating the dislocations propagation under strong size and interface constraints, Figure 10 illustrates the dislocations slip processes in Ni layer with *d* = 25.0 nm. The upper interface and FCC atoms have been eliminated to better observe dislocations propagation at different size constraints.

Figure 10a displays the morphology of extended dislocations in Ni layer for the case of *λ* = 1.31 nm. The extended dislocations are defined as defect systems consisting of two partial dislocations with a segment of stacking fault between them. As displayed in the enlarged illustration in Figure 10a, leading dislocation with $b_{l}=1/6[\bar{1}2\bar{1}]$ first emits from the GBs, the dislocation sliding is confined and move along the (111) plane in Ni layer. Subsequently, trailing dislocation with $b_{t}=1/6[11\bar{2}]$ also emits rapidly from the GBs and propagates along the same (111) plane. The angle between two partial dislocations is 60°, and the width of the stacking fault remains invariable throughout the slip process. Extended dislocations slide along specific directions and are absorbed by the opposite GB eventually. For the case of the tiny layer thickness, dislocations emitting from GBs tend to move by forming extended dislocations as the interface dislocations are completely suppressed. This explains why extended dislocations are observed during the tensile process of all Ti/Ni PNLs with *λ* = 1.31 nm. However, it is noted that motion of extended dislocations requires the simultaneous displacement of atoms near partial dislocations and in the stacking fault segment, which leads to significant increase in energy barrier for slip [57].

As the layer thickness increases, interface dislocations have ample space to nucleate and move in Ni layer. Thus, GB and interface can act synergistically to facilitate dislocations motion at this condition. As shown in Figure 10b,c, the initial dislocations generate from the GBs and move in Ni layer under the interface constraint. Partial dislocations propagating parallel to the interface changes the local interfacial structure, which promotes the nucleation of interface dislocations at the junction regions. And the interface dislocations move toward the opposite side of the interface under the external loading. Since both interfacial dislocations and dislocations emitting from grain boundaries propagate on the same slip plane, the stacking faults generated by moving dislocations are truncated upon intersection. This interaction leads to the formation of pairwise dislocations or new partial dislocations. For Ti/Ni PNLs with *λ* > 1.31 nm, the ample space guarantees the nucleation and propagation of interface dislocations. Both GB and interface can promote nucleation and growth of the moving dislocations. Consequently, GB and the interface will jointly govern dislocation propagations in Ni layer.

**Figure 11:**
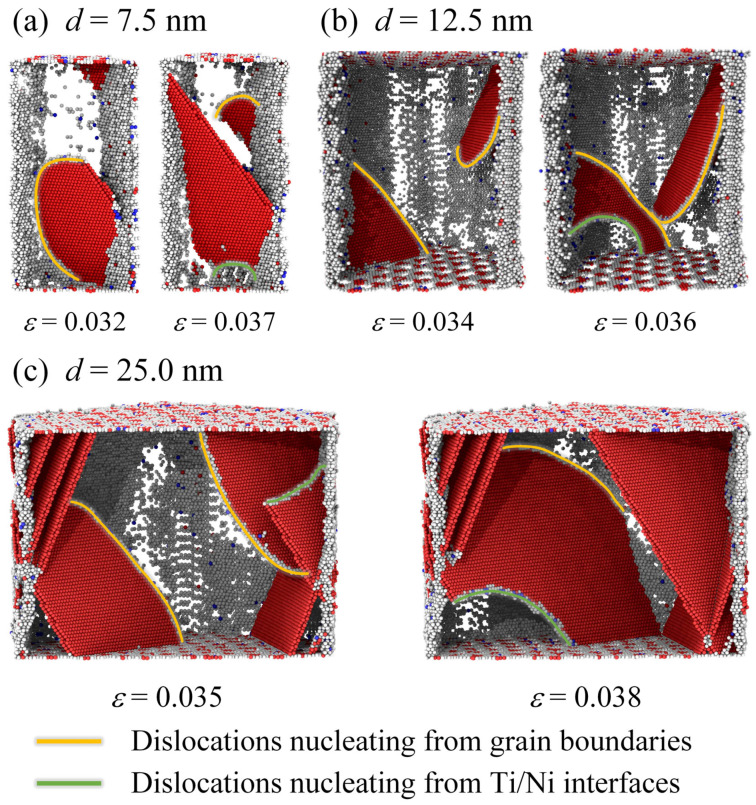
Dislocations propagation in Ni sublayer under grain boundary and interface constraints for the polycrystalline Ti/Ni nanolaminate with *λ* = 15.15 nm. (**a**) *d* = 7.5 nm, (**b**) *d* = 12.5 nm, (**c**) *d* = 25.0 nm. The FCC atoms have been removed to better observe defect evolutions.

Further assessing the synergistic effect of GB and interface on the dislocation motions in Ni layer, Figure 11 illustrates the dislocations propagation at the grain interior of Ti/Ni PNLs with *λ* = 15.15 nm. For clarity, the FCC atoms inside the grain and some boundary atoms have been removed. The orange lines in Figure 11 represent dislocations nucleating from GBs, while the green lines represent dislocations nucleating from the interfaces. Owing to severe stress concentration at the intersection of interface and grain boundary, dislocations first nucleate from these regions and are confined to move within the grain until they are absorbed by a nearby interface or GB. The dislocations sliding along the interface disrupt the congruence of the interface, making it easier to nucleate defects. As the external load is applied, trailing dislocations first nucleate near the interface and tend to move along the same plane as the leading dislocations. The movement of interface dislocations causes the stacking faults to gradually disappear. Dislocations emitting from GB and interface can also form pairwise dislocations. Unlike the extended dislocations formed with tiny sublayers, the angle between these partial dislocations is not fixed. Furthermore, this structure quickly disappears as the interface dislocations reach the GB or the opposite interface, whereas extended dislocations can slide stably within the Ni sublayer. Therefore, both GB and interface can promote the nucleation and propagation of dislocations, while also hinder or absorb dislocations moving toward them. They can serve as dislocation sources and dislocation sinks during the plastic deformations [58,59]. This synergistic effect enables Ni layer to withstand greater plastic deformations.

### 4.5. Constrained Plastic Deformations of Ti/Ni Polycrystalline Nanolaminates

As depicted by the preceding analysis, the plastic deformations of Ti/Ni PNLs are conducted through the plastic co-deformation of the constituent phases under strong constraint of size and interface. The plastic deformations of the different phases, especially Ni sublayer, are jointly determined by layer thickness and grain size. Therefore, a dimensionless parameter of *d*/*λ* accounting for grain morphology and interface structure may better reflect the controlling factors of the deformation mechanisms.

**Figure 12:**
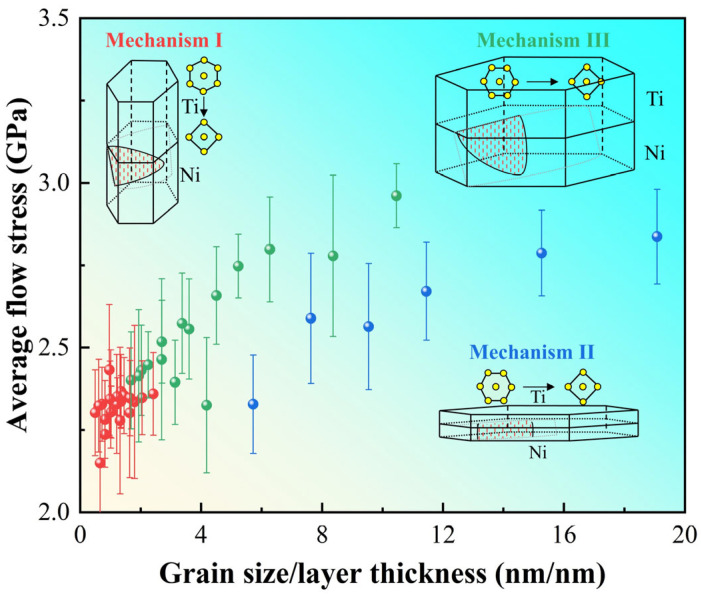
The relationship between average flow stress and the ratio of grain size to layer thickness (*d*/*λ*) for different Ti/Ni polycrystalline nanolaminates. Error bars indicate the standard deviation. Three typical plastic deformation mechanisms are also shown in the figure.

As shown in Figure 12, the flow stress shows three distinct trends with increasing *d*/*λ*, which corresponds to the three deformation mechanisms within the grains. Among these mechanisms, the plastic deformations of Ti layer are size-independent and always manifest as HCP-BCC phase transformations. For the case of d/λ<<1, the flow stresses remain constant values or increases slightly with increasing *d*/*λ*. Under these conditions, the grain shows an elongated columnar morphology. Plasticity in the Ni layer is primarily controlled by grain size, manifesting as confined intragranular dislocation slip, as illustrated by Mechanism I in Figure 12. For the case of d/λ>>1, the flow stresses exhibit two distinct trends with increasing *d*/*λ*. Compared to the values of samples with *λ* = 1.31 nm, the flow stresses of samples with *λ* > 1.31 nm shows a steeper increase as *d*/*λ* increases. When the samples are with *λ* = 1.31 nm, the strong constraint of the interface leads to grain size variations that rarely alter the plastic deformation carriers, and the plastic deformations in Ni layers are propagation of extended dislocations, as shown by mechanism II. When the samples are with *λ* > 1.31 nm, the grains become flattened, and plastic deformations are primarily controlled by the layer thickness, which manifests as dislocation-confined layer slip, as shown by mechanism III. Except for mechanism II, the other two mechanisms may dominate the plastic deformations of the material collectively, especially in the case when grain size and layer thickness are comparable. It should be noted that the flow stress is related to plastic deformation of different components, such as crystalline phases, GB, and interface. When evaluating the overall plasticity of the Ti/Ni PNLs, the contribution of intragranular deformations to the overall plasticity of the materials must be considered, along with that of plastic deformations at GB and interface. These plastic deformations at GBs and interfaces are also determined by the feature size. The foregoing analysis suggests that grain boundary plasticity becomes increasingly pronounced at smaller grain sizes. Conversely, interface plasticity gains significance with reduced layer thickness. This can also explain the three distinct trends of the flow stresses.

In summary, the overall plastic deformations of Ti/Ni PNLs are primarily driven by plastic deformations of the grains, which shows different deformation patterns under the strong size and interface constraints. GB and interface participate in the overall deformations through their own plastic deformations. When the space within grain or layer is very narrow (*d* >> *λ* or *d* << *λ*), the interfaces or grain boundaries transform from passive obstacles into structural units that are actively involved in plastic deformations [52]. Furthermore, GB and interface also act to coordinate the deformations of different phases and grains during the tensile process. This coordination effect endows the material with relatively favorable plastic properties.

This study mainly focuses on the size-and interface-constrained tensile behavior of Ti/Ni PNLs. Actually, grain distribution, interface morphology, and stress states (tension, compression, shear, and nanoindentation, etc.) may influence the mechanical behavior of the materials. Further investigation is needed to determine how these factors affect the deformation behavior of the nanolaminates. Furthermore, due to the inherent limitations of molecular dynamics simulations, simulations at larger scales (submicron or micron scales) require enormous computational resources. There is an urgent need to conduct research on the multiscale mechanical behavior of Ti/Ni nanolaminates. Future research could focus on establishing a multiscale simulation framework to gain a more comprehensive understanding from atomic-scale nucleation to microstructure evolution. For example, the atomic-scale information on interfacial properties and dislocation behavior obtained in this study could serve as key input parameters for mesoscale models such as the phase-field method (PFM). This approach has already been used to simulate structure evolution and predict material properties on larger spatiotemporal scales. Such studies have fully demonstrated the feasibility of integrating computational data into mesoscale models to predict complex interfacial behavior [60]. Hence, extending this methodology to the simulation of mechanical behavior and performance of nanolaminated materials represents a promising and worthwhile direction for future research. Moreover, although MD methods offer significant advantages in revealing the underlying deformation mechanisms of the materials, and can qualitatively predict trends in the mechanical properties of the materials, there remains a considerable discrepancy between MD results and experimental data. This discrepancy is primarily due to the vast disparity between the scale of the computer-made simulations and strain rates. Therefore, more work needs to be done by strain rate scaling analyses or accelerated methods to bridge this gap and gain a more systematic understanding of the deformation mechanisms of the materials.

## 5. Conclusions

MD method was employed to investigate the effect of grain size *d* (7.5~25.0 nm) and layer thickness *λ* (1.31~15.15 nm) on the tensile behavior of Ti/Ni polycrystalline nanolaminates. Moreover, the roles of the Ti sublayer, Ni sublayer, grain boundary, and interface during the plastic deformations were assessed in this work. The main conclusions are as follows:

(1) Variations in grain size and layer thickness rarely affect yield stresses of Ti/Ni PNLs. The yield stresses of most Ti/Ni PNLs remain consistent except the value of the sample with *d* = 7.5 nm and *λ* = 1.31 nm. In contrast, the plastic properties of Ti/Ni PNLs are strongly related to the feature size, and the nanolaminates possess sustained high-flow stress under the conditions of the smaller layer thickness and larger grain size.
(2) Plastic deformations of Ti/Ni PNLs are dominated by the plastic co-deformation of different structures under strong size and interface constraints. The dominant plastic deformations in Ti layer are phase transformation, which is independent of grain size and layer thickness. Dislocations propagation in Ni layer is highly dependent on feature size. For the sample with *λ* = 1.31 nm, the principal plastic deformation is the formation and gliding of extended dislocations. For the sample with *λ* > 1.31 nm, the dominated plastic deformation is the interaction between moving dislocations and interface dislocations. Moreover, interface migration, grain boundary diffusion, and grain boundary migration become prominent plastic deformation carriers in the case of small grain sizes or layer thicknesses. The coordinating effect of grain boundary and interface on deformation between different structures endows the Ti/Ni PNLs with relatively favorable plastic properties.
(3) The flow stresses of Ti/Ni PNLs are closely related to the dimensionless parameter *d*/*λ*, and the parameter accounting for grain morphology and interface structure can characterize the controlling factors of different deformation mechanisms effectively. For the case of d/λ<<1, the flow stresses remain in constant value or increases slightly with increasing *d*/*λ*. Plastic deformations within Ni layer are controlled by the mechanism of dislocation-confined intragranular slip. For the case of d/λ>>1, the flow stresses exhibit two distinct rising tendencies with increasing *d*/*λ*. When the samples with *λ* = 1.31 nm, plastic deformations in Ni layers are dominated by the mechanism of extended dislocation-confined layer slip. For the larger layer thickness, plastic deformations are controlled by the mechanism of dislocation-confined layer slip.

## Data Availability

The original contributions presented in this study are included in the article. Further inquiries can be directed to the corresponding authors.

## Supplementary Materials

The following supporting information can be downloaded at https://www.mdpi.com/article/10.3390/nano16100588/s1, Figure S1. Equilibrium lattice constants and cohesive energies of (a) Ti and (b) Ni. Table S1. Equilibrium lattice constants and cohesive energies of Ti and Ni. Figure S2. Generalized stacking fault energy of Ni along [112] direction. Figure S3. Variations in potential energy of Ti/Ni PNL with *d* = 7.5 nm and *λ* = 1.31 nm during the relaxation process. Figure S4. Tensile properties of Ti/Ni PNL. (a) Stress–strain curves, (b) Average flow stress. Refs. [32,33,61,62,63,64,65,66,67,68,69] are cited in the supplementary materials.

## Abbreviations

The following abbreviations are used in this manuscript:

PNLs	Polycrystalline Nanolaminates
MNLs	Metallic Nanolaminates
FCC	Face-Centered Cubic
BCC	Body-Centered Cubic
HCP	Hexagonal Close-Packed
MD	Molecular Dynamics
CNA	Common Neighbor Analysis
DXA	Dislocation Extraction Algorithm
GB	Grain Boundary
