# Supplementary material for: Size- and Interface-Constrained Tensile Behavior of Ti/Ni Polycrystalline Nanolaminates: Insight from Molecular Dynamics
Source: Nanomaterials (Basel). 2026 May 12;16(10):588. doi: 10.3390/nano16100588 (PMC13209561; doi:10.3390/nano16100588)
Supplement: Supplementary file 1 [file nanomaterials-16-00588-s001.zip › nanomaterials-4278459-supplementary.pdf]

Supplementary Information

Size- and Interface-Constrained Tensile Behavior of Ti/Ni Polycrystalline Nanolaminates: Insight from Molecular Dynamics

Mengjia Su[1, 2,*], Lanting Liu[3], Wei Hu[4,5], Qiong Deng[1,2,**]

1 School of Aeronautics, Northwestern Polytechnical University, Xi'an 710072, China

2 National Key Laboratory of Strength and Structural Integrity, Xi'an 710072, China

3 School of Science, Chang'an University, Xi'an 710064, China

4 School of Flight Technology, Jiangxi Flight University, Nanchang 330088, China

5 Key Laboratory of Low Altitude Geographic Information and Air Route of Jiangxi Education Institutes, Nanchang 330088, China

* Corresponding authors: sumj_sy@nwpu.edu.cn (Mengjia Su)

dengqiong24@nwpu.edu.cn (Qiong Deng)

1. Verification of potential for lattice properties of Ti and Ni

To demonstrate the validation of the algorithm and simulation method, we have calculated the equilibrium lattice constants and corresponding cohesive energies of monocrystalline Ti and Ni by using the EAM potential proposed by Zhou and Wadley [1, 2], and compared with those values obtained by using the MEAM potentials [3, 4]. Figure 1 shows the equilibrium lattice constants and corresponding cohesive energies calculated by different potentials. As shown in Figure S1(a), the equilibrium lattice constant of HCP-Ti calculated by EAM potential is about 2.948 Å and cohesive energy is -4.868 eV/atom. While equilibrium lattice constants by other MEAM potentials are 2.949 and 2.948 Å, corresponding cohesive energies are -4.872 and -4.869 eV/atom, respectively. It should be noted here that the c/a ratio in our simulation is set as

1.587. Figure S1(b) shows the equilibrium lattice constant of FCC-Ni calculated by EAM potential is about 3.520 Å, and cohesive energy is -4.449 eV/atom. While equilibrium lattice constants by other MEAM potentials are 3.521 and 3.523 Å, corresponding cohesive energies are -4.449 eV/atom, respectively. The above calculated values are listed in Table S1.

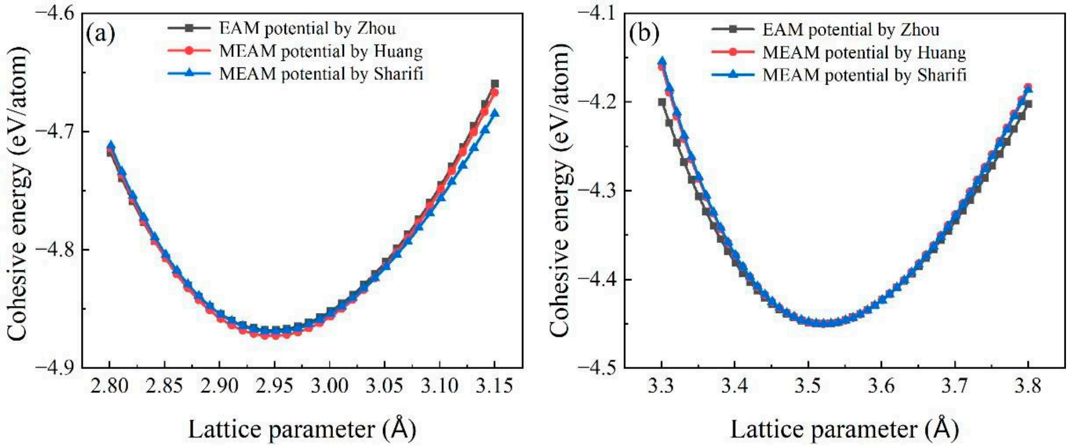

Figure S1. Equilibrium lattice constants and cohesive energies of (a) Ti and (b) Ni.

Table S1. Equilibrium lattice constants and cohesive energies of Ti and Ni

Element	Methods	Lattice constant (Å)	Cohesive energy (eV/atom)
Ti	EAM [1, 2]	a = 2.948, c = 4.678	-4.868
	MEAM [3]	a = 2.949, c = 4.680	-4.872
	MEAM [4]	a = 2.948, c = 4.678	-4.869
	Other works	a = 2.950, c = 4.683 [5]	4.85 [6]
Ni	EAM [1, 2]	3.520	
	MEAM [3]	3.521	-4.449
	MEAM [4]	3.523	
	Other works	3.524 [7]	-4.450 [8]

As shown in Figure S1 and Table S1, the calculated fundamental properties of Ti and Ni by using the EAM potential show good agreement with experimental and DFT results [5-8], which confirms the reliability of the potential for describing the individual phases.

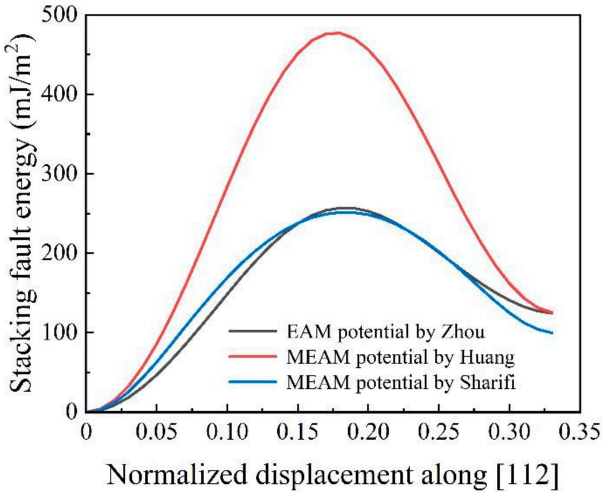

Figure S2. Generalized stacking fault energy of Ni along [112] direction.

To address the plastic behavior at Ni layer, the generalized stacking fault energy (GSFE) curves for the <112> slip systems have been calculated, as shown in Figure S2, in which the nucleation and propagation of dislocations are always discovered in Ni layer. As shown in Figure S2, the unstable stacking fault energy (γ_{usf}) obtained from EAM potential by Zhou is about 257.09 mJ/m^2, and the intrinsic stacking fault energy (γ_{isf}) is 124.54 mJ/m^2. Those values are in good agreement with the experimental and DFT results. In this work, the γ_{usf} calculated by the DFT method ranges from 230 to 305 mJ/m^2, and γ_{isf} ranges from 110 to 140 mJ/m^2 [8-10], and the γ_{isf} obtained by the experiment ranges from 110 to 135 mJ/m^2 [8, 9, 11]. However, the γ_{usf} calculated by the MEAM potential proposed from Huang (476.96 mJ/m^2) is significantly higher than the values obtained from the other two potentials, while the γ_{isf} obtained by the MEAM potential proposed from Sharifi (99.746 mJ/m^2) is slightly lower than the values obtained from the other two potentials.

Therefore, the unstable stacking fault energy and intrinsic stacking fault energy calculated using EAM potential are consistent with the values reported in the literature. This demonstrates the effectiveness of the potential in describing plastic deformation in Ni layer.

2. Verification of effectiveness of simulation method for the tensile behavior of Ti/Ni PNLs

To validate the effectiveness of the simulation method for the tensile behavior of Ti/Ni PNLs, three replicated tensile tests for the Ti/Ni PNL with d = 7.5 nm and λ = 1.31 nm were performed. The loading conditions in all three simulations remained consistent, and the only difference was the value of random seed used in the LAMMPS code.

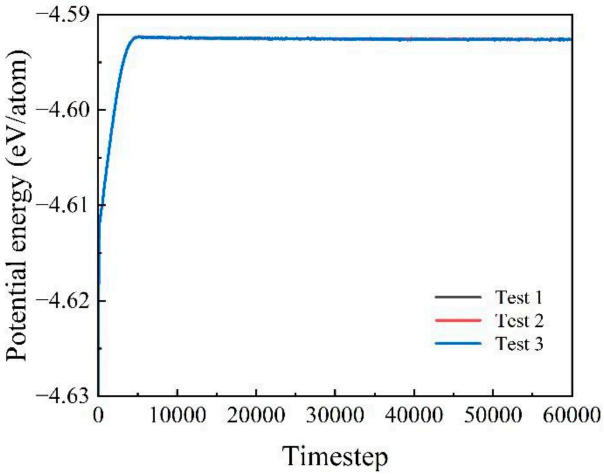

Figure S3. Variations in potential energy of Ti/Ni PNL with d = 7.5 nm and λ = 1.31 nm during the relaxation process.

Figure S3 shows the variations in potential energy of Ti/Ni PNL during the relaxation process. As can be seen from the Figure S3, the three individual potential energy curves are nearly identical, which demonstrates the effectiveness of our method in studying the relaxation properties of Ti/Ni polycrystalline nanolaminate.

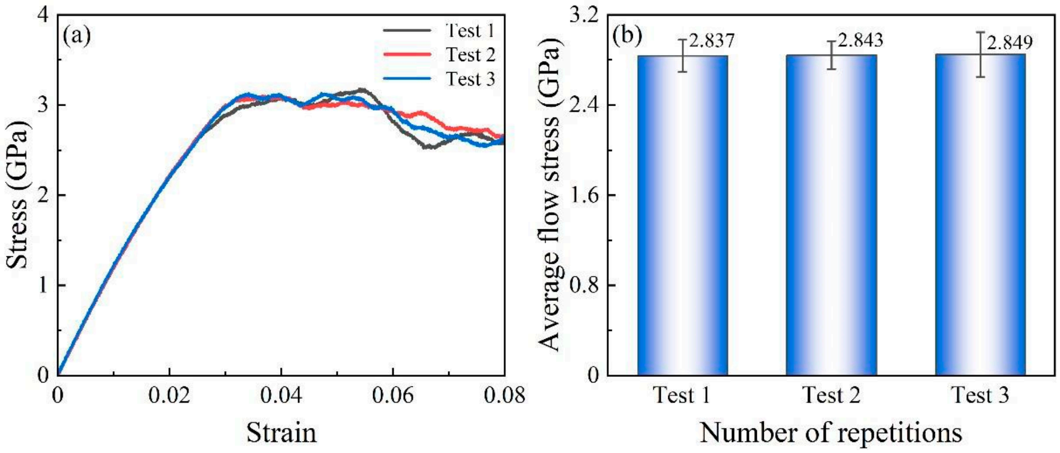

Figure S4. Tensile properties of Ti/Ni PNL. (a) Stress–strain curves, (b) Average flow stress.

Figure S4 shows the tensile properties of Ti/Ni PNL with d = 7.5 nm and λ = 1.31 nm. It is clear that the initial elastic stages of the stress–strain curves for the three tests largely coincide.

After entering the plastic flow stage, the three curves fluctuate around the specific values or decline slightly, as shown in Figure S4(a). A further analysis of the plastic flow stress during the three tensile tests reveals that the average flow stress remained essentially constant across the three simulations, as shown in Figure S4(b). Thus, Figure S4 confirms the consistency of our algorithm and simulation method in describing the mechanical behavior of Ti/Ni PNLs.

References

[1] Wadley H.N.G., Zhou X.W., Johnson R.A., et al. Mechanisms, models and methods of vapor deposition[J]. Progress in Materials Science, 2001, 46(3-4): 329-377.

[2] Zhou X.W., Wadley H.N.G., Johnson R.A., et al. Atomic scale structure of sputtered metal multilayers[J]. Acta Materialia, 2001, 49(19): 4005-4015.

[3] Huang S., Xiong Y., Ma S., et al. Enhancing the irradiation resistance of $L1_2$ intermetallics by incorporating multiple principal elements through computational modeling[J]. Journal of Materials Research and Technology, 2024, 30: 9274-9284.

[4] Sharifi H., Wick C.D. Developing interatomic potentials for complex concentrated alloys of Cu, Ti, Ni, Cr, Co, Al, Fe, and Mn[J]. Computational Materials Science, 2025, 248: 113595.

[5] Clark Jr H.T. The lattice parameters of high purity alpha titanium; and the effects of oxygen and nitrogen on them[J]. JOM, 1949, 1(9): 588-589.

[6] Kittel C., McEuen P. Introduction to solid state physics[M]. John Wiley & Sons, 2018.

[7] Arblaster J.W. Selected values of the crystallographic properties of elements[M]. ASM International, 2018.

[8] Gong X., Li Z., Pattamatta A.S.L.S., et al. An accurate and transferable machine learning interatomic potential for nickel[J]. Communications Materials, 2024, 5(1): 157.

[9] Zhao S., Stocks G.M., Zhang Y. Stacking fault energies of face-centered cubic concentrated solid solution alloys[J]. Acta Materialia, 2017, 134: 334-345.

[10] Shang S.L., Gao M.C., Liu Z.K. Temperature-Dependent Mechanical Properties of Ni-Based Concentrated Alloys: Insights from First-Principles Calculations[J]. High Entropy Alloys & Materials, 2025, 3(2): 307-321.

[11] Smallman R.E., Dillamore I.L., Dobson P.S. The measurement of stacking fault energy[J]. Le Journal de Physique Colloques, 1966, 27(C3): 86-93.
